# Efficacy of preoperative Tranexamic Acid in patients undergoing intertrochanteric hip fracture surgery: A randomized placebo controlled trial

**DOI:** 10.12669/pjms.39.6.7667

**Published:** 2023

**Authors:** Faaiz Ali Shah, Naeemullah, Mian Amjad Ali, Mian Javed Iqbal

**Affiliations:** 1 Faaiz Ali Shah, FCPS, Orthopaedics & Traumatology Division Lady Reading Hospital, Peshawar, Pakistan; 2 Naeemullah, FCPS, Orthopaedics & Traumatology Division Lady Reading Hospital, Peshawar, Pakistan; 3 Mian Amjad Ali, PhD, Orthopaedics & Traumatology Division Lady Reading Hospital, Peshawar, Pakistan; 4 Mian Javed Iqbal, FCPS, Orthopaedics & Traumatology Division Lady Reading Hospital, Peshawar, Pakistan

**Keywords:** Dynamic Hip Screw, Hemoglobin, Intertrochanteric, Transfusion, Tranexamic Acid

## Abstract

**Objective:**

To determine the efficacy of preoperative Tranexamic Acid (TXA) in reducing perioperative allogenic blood transfusion frequency in patients with intertrochanteric fractures treated with Dynamic Hip Screw (DHS).

**Methods:**

We conducted this placebo controlled double-blinded randomized trial in Orthopaedics & Traumatolgy Division Lady Reading Hospital, Peshawar from 1^st^ January 2020 to 25^th^ December 2022. All patients with intertrochanteric hip fractures fulfilling the inclusion criteria were treated with Dynamic Hip Screw (DHS) and were randomly divided into two equal groups. One group was administered single dose IV Tranexamic Acid (TXA) in a dose of 15mg/kg body weight in 100ml of saline while the other group (Placebo) was given 100 ml normal saline preoperatively. Post-operative Hemoglobin was measured on first, second and third day. The frequency of allogenic blood transfusions in the perioperative period was determined in both groups based upon the transfusion trigger (Hemoglobin 9g/dl). Categorical variables were compared with Chi-square test and mean with Independent sample t-test. *P* value of <0.05 was considered significant.

**Results:**

The total number of patients in our trial were 200 which were equally but randomly allocated into TXA group and Placebo group each containing 100 patients. The mean age of the patients in TXA group was 48.16±1.75 years and the age of patients in the Placebo group was 48.35±1.60. The baseline demographic and clinical variables of both groups were identical (*p*< 0.05). The average preoperative hemoglobin was 11.5±4.2 g/dl in TXA group and 11.3±2.4g/dl in the Placebo group (p>0.05). The frequency of allogenic blood transfusion was significantly lower (*p*<0.05) in TXA group (13%, n=13) than in the Placebo group (41%, n=41).

**Conclusion:**

Tranexamic acid (TXA) significantly reduces the frequency of peri operative allogenic blood transfusion in patients undergoing Dynamic Hip Screw (DHS) for intertrochanteric fractures.

## INTRODUCTION

Intertrochanteric fractures account for about half of all hip fractures.^1^ These fractures are usually treated with Dynamic Hip Screw (DHS) and Proximal Femoral Nail Antirotation (PFNA) nail.^2^ In addition to the intraoperative visible blood loss hidden blood loss which is often underestimated but may measure up to 1500 ml has been reported in intertrochanteric fractures and resulting in peri operative blood transfusion in 20% to 60% of thesez patients.^3^,^4^ Patients with perioperative allogenic blood transfusion have longer hospital stay, increased cost and are more prone to transfusion related adverse reactions, post-operative bacterial infections and cardiac complications.^5^-^7^

Post-operative low hemoglobin level however is one of the risk factor for low functional outcome and higher mortality rates in patients with intertrochanteric fractures.^8^ In order to improve the surgical outcome and reduce the post-operative complications of intertrochanteric fractures the perioperative blood loss must be reduced.^4^ To minimize perioperative blood loss various methods have been used including minimally invasive surgical approach, optimum intraoperative hemostasis, controlled hypotension, salvage of blood during surgery and usage of perioperative antifibrinolytic agents.^9^ Tranexamic acid (TXA) is a simple, inexpensive and readily available antifibrinolytic agent which consists of synthetic analogue of Lysine amino acid that binds to Plasminogen receptors and stabilizes the blood clot and reduces bleeding.^10^

Peri operative Tranexamic acid (TXA) has been shown to reduce bleeding and blood transfusion rates in total knee, total hip, spine surgery and intertrochanteric fractures with nailing but its efficacy in intertrochanteric fractures treated with dynamic Hip Screw (DHS) is still controversial.^11^,^12^ The objective of our study was to determine the efficacy of preoperative TXA in reducing perioperative allogenic blood transfusion frequency in patients with intertrochanteric fractures treated with DHS. We hypothesized that single intravenous injection of TXA before induction will significantly reduce the perioperative blood loss and lower the frequency of allogenic blood transfusion. The results of our study will be useful to formulate standard guidelines for the routine use of TXA in DHS surgery.

## METHODS

We conducted this placebo controlled double-blinded randomized trial in Orthopaedic and Traumatology Division Lady Reading Hospital Peshawar Pakistan from 1^st^ January 2020 to 25^th^ December 2022. This trial was approved by Ethical Review Board Lady Reading Hospital Medical Teaching Institution (Approval No.347/LRH). The trial was prospectively registered with Australian New Zealand Clinical Trial Registry (ANZCTR) with trial registration no. ACTRN1262000105994.

We drafted our study protocol and then conducted and reported our trial in accordance with Consolidated Standard of Reporting Trials (CONSORT)^13^ We calculated our trial sample size with World Health Organization(WHO) sample size calculator keeping the level of significance(**α**) **at** 5% and power of test(1-**β**) **at** 90% and based upon 17% transfusion requirement in TXA(P_1_) versus 35% in normal saline Placebo(P_2_)^4^. Our sample size was 200 equally divided into TXA group and Placebo group with 100 patients each. Non probability consecutive sampling technique was adopted.

We included patients of both gender, 18 years and above with simple two part intertrochanteric fracture (AO 31A1.2/31A1.3) with Hemoglobin ≥10 g/dl and presented within one week of sustaining the fracture. We excluded all those patients who had pathological fractures, open fracture, multiple fractures, history of Deep Venous Thrombosis (DVT), Pulmonary Embolism (PE), stroke and Myocardial Infarction in the past one year. Similarly, patients with hepatorenal dysfunction, Disseminated Intravascular Coagulation, allergy to TXA, using anticoagulants, body weight more than 100 kg, pregnant and lactating females and those who had received pre surgery transfusion were also excluded. Written informed consent was obtained from all study participants. All patients were administered 40mg Enoxaprin (Clexane® SANOFI) subcutaneously once daily for prophylaxis against DVT 12 hours before surgery and then 72 hours after surgery for seven days.

We randomized our patients into two groups to received either intravenous Tranexamic Acid (TXA group) or normal saline(Placebo group).This allocation was done randomly with the help of computer generated random numbers by a third party(ward clerk) who was not part of this trial and who maintained the secrecy throughout the conduct of this trial. To ensure masking identical drips simply labelled as “Trial Drug” were used for both TXA group and Placebo group and were administered by nursing staff. Tranexamic Acid (Injection TRANSAMIN® 500gmg, Hilton Pharma) was used in a dose of 15mg/kg body weight in 100ml of saline while in Placebo group 100ml of normal saline (Tran-Sol-NS®, APL) was administered over 10 minutes before induction of anaesthesia.

The principal author of this trial fixed all the fractures with DHS under spinal or general anaesthesia on traction table and under image intensifier. A uniform standard opens operative technique and intra operative management of hypotension and fluid replacement technique as advised by Chen et al.^4^ was adopted for all the surgeries. Hemoglobin was measured on admission and before the induction of anaesthesia. Post operatively hemoglobin was measured on first, second and third post-operative day or after every transfusion. Post operatively all patients were assessed at two weeks initially in OPD and then once a month thereafter for 12 weeks. The assessment was done by a senior Orthopaedic consultant who was not part of trial team. In each visit X-ray was done to assess the fracture healing while clinical examination was performed to document any complication. The frequency of allogenic blood transfusion in the perioperative period was determined in both groups based upon the transfusion trigger (Hemoglobin 9g/dl).

We analyzed the data of our trial with the help of SPSS version 24. Mean±Standard Deviation (SD) was calculated for continuous variables while frequencies and percentages for categorical variables. Categorical variables and means were compared with Chi-square test and independent sample-t test respectively. A *P-value* of <0.05 was considered significant. Data was presented in tables and flow chart where appropriate.

## RESULTS

The total eligible patients with intertrochanteric fractures were 271. After the application of our exclusion criteria 200 patients consented for the trial and were enrolled. All patients were randomly divided into Tranexamic acid (TXA) and Placebo (normal saline) group each containing 100 patients. The mean age of TXA group was 48.16±1.75 (range 31 years to 83 years) while the mean age of Placebo group was 48.35±1.60 (range 32 years to 82 years). There was no statistically significant difference in the baseline demographic and clinical variables of both groups (*p*< 0.05) as shown in Table-I.

**Table-I:** Comparison of baseline demographic and clinical variables of both groups.

Demographic/clinical Variables	TXA(n=100)	Placebo(n=100)	P value
Age±SD(years)	48.16±1.75	48.35±1.60	0.83
*Gender*	N (%)	N (%)	
Male	72(72%)	70(70%)	0.12
Female	28(28%)	30(30%)	0.32
***Fracture side***			
Right side	59(59%)	58(58%)	0.09
Left side	41(43%)	42(44%)	0.07
***Aetiology of Intertrochanteric fractures***			
Fall	43(43%)	46(46%)	0.34
Motorbike Accident	32(32%)	30(30%)	0.44
Motor Vehicle Accident	25(25%)	24(24%)	0.23
***Types of Intertrochanteric fractures***			
OTA/AO 31A1.2	65(65%)	63(63%	0.06
OTA/AO 31A1.3	35(35%)	37(37%)	0.08

The mean preoperative hemoglobin prior to induction of anaesthesia was 11.5±4.2 g/dl in TXA group and 11.3±2.4g/dl in the Placebo group (p>0.05). Hemoglobin on post-operative day one and two revealed a statistically significant drop (*p*<0.05) in Placebo group (8.3±1.7 g/dl and 9.5±2.1g/dl) than in TXA group (9.4±1.5 g/dl, 10.3±1.3g/dl) as shown in Table-II. The frequency of allogenic blood transfusion was significantly reduced (p<0.05) in TXA group (13%, n=13) than in the Placebo group (41%, n=41) both intra operatively as well as postoperatively. The TXA group were transfused 1.3 packs per patient while placebo group were transfused on average 3.2 packs (*p*<0.05). Stratification of data for age, gender, side, aetiolgy and type of fracture did not show any significant difference in frequency of allogenic blood transfusion. (*p*>0.05)

**Table-II:** Comparative analysis of pre and post-operative hemoglobin levels and transfusion rates.

S.no	Hematological data variables		TXA(n=100)	Placebo(n=100)	P value
1	Hemoglobin(g/dl)	On admission	11.4±3.1	11.1±2.2	0.21
		Prior to surgery	11.5±4.2	11.3±2.4	0.32
		Postoperative day one	9.4±1.5	8.3±1.7	0.001
		Postoperative day two	10.3±1.3	9.5±2.1	0.002
		Postoperative day three	10.4±2.2	10.1±1.3	0.11
2	Number of patients requiring Allogenic blood transfusion packs	Intraoperative	04(4%	11(11%)	0.01
		Post-operative	09(9%)	30(30%)	0.007
		Total	13(13%)	41(41%)	0.005
3	Mean number of units of packs transfused per patient		1.3(1 to 2)	3.2(2.2 to 4.1)	0.001

The overall complications were noted in 09(9%) patients in the TXA group and 11(11%) patients in the Placebo group at 12^th^ week of follow up. The frequency of post-operative complications in both groups however had no statistically significant difference (*p*>0.05) as shown in Table-III.

**Table-III:** Comparison of post-operative complications in both groups.

S. No.	Complications	TXA(n=100)	Placebo(n=100)	P- value
1	Transfusion reaction	02	03	0.82
2	Superficial surgical site infection	02	02	0.32
3	Hematoma formation	01	01	0.14
4	Deep Venous Thrombosis (DVT)	01	01	0.40
5	Pulmonary Embolism	01	01	0.20
6	Myocardial Infarction	00	01	0.90
7	Stroke	01	00	017
8	Death	01	02	0.86

## DISCUSSION

In our trial we had documented that pre-operative administration of Tranexamic acid (TXA) to intertrochanteric fractures treated with DHS resulted in significantly lower frequency of perioperative allogenic blood transfusion than the Placebo. When we searched the literature, we found few relevant international trials and only one national or local trial favoring our results although minor differences in study methodology like different types of hip fracture surgeries, mean age of the patients, dosage and frequency of TXA administration, transfusion threshold and follow up period exist.

Haj-Younes et al.^14^ conducted a systematic review and meta-analysis of 10 randomized controlled trials comprised of 842 patients for assessing the efficacy of pre-operative TXA involving hip arthroplasty, nailing and DHS surgery. They reported that very a smaller number of patients required peri operative blood transfusion in TXA group (RR 0.72, 95% CI, 0.59-0.88) than in the control group. Amer et al.^15^ conducted a meta-analysis and systematic review of 559 patients in seven studies involving Hemiarthroplasty, Total Hip Replacement (THR), nailing and DHS. The data revealed a significant reduction of perioperative allogenic blood transfusion frequency in TXA than control (RR 0.54, 95% CI, 0.726, *p*<0.001).

Dynamic hip screw surgery is an emergency trauma surgery. It is different from elective hip surgery. Open surgical technique is usually adopted for performing DHS surgery. Tourniquet application which can reduce bleeding is not possible in this surgery. Excessive peri operative blood loss resulting in allogenic whole blood transfusions are therefore more in DHS surgery. In our set up whole blood is more frequently used in DHS surgery because it is promptly available and economically feasible in emergency. In our study the frequency of allogenic blood transfusion in the TXA group was 13%(n=13) when compared to in the Placebo group 41%(n=41). Similar to our trial Baruah et al.^16^ randomly administered TXA or normal saline to 30 patients each undergoing DHS surgery for intertrochanteric fractures. The transfusion threshold was Hemoglobin 8.5 g/dl (Hematocrit <27%). Intra operative blood transfusion frequency was 90%(n=27) in TXA group versus 100%(n=30) in the control group.(*p*<0.001) The frequency of post-operative blood transfusion was 20%(n=6) in the TXA group and 50%(n=15) in the control group.(p<0.014). In one retrospective multicenter study the data of 159 patients with Hemiarthroplasty, THR, nailing and DHS were analyzed by Geddes^17^ and reported that transfusion frequency was significantly lower (*p*<0.05) in the TXA group (6.52%, n=3/46) than the control (20.35%, n=23/113).

Zufferey et al.^18^ conducted a double blinded placebo trial of 57 patients who were treated with THR (40%, n=23), DHS (37%, n=21), nailing (21%, n=12) and Hemiarthroplasty (2%, n=1). They documented that the frequency of blood transfusion was 42% in TXA group versus 60% in placebo group (*p*>0.05). AlSumadi^19^ performed a comparative study of 613 patients treated with Hemiarthroplasty (47.6%) DHS (34.9%), THR (8.5%), cannulated screws (1%) and nailing (7%). Results showed that blood transfusion frequency was statistically lower (*p*<0.05) in TXA group (13%, n=41) than in control (28%, n=85). These authors concluded that TXA is effective in reducing transfusion rates in patients with hip fracture surgeries including DHS surgery.

In one national study conducted in Agha Khan University Hospital Karachi Pakistan by Mohib^20^ compared 100 patients of intertrochanteric fractures who received either two doses intravenous TXA 10 mg/kg body weight or normal saline preoperatively and three hours after surgery. The mean age of TXA group was 69.0±10.0 years and control group were 70±9.4 years. The allogenic blood transfusion indication was hemoglobin <7g/dl measured 24 hours postoperatively. These authors reported blood transfusion in 9(18%) patients in TXA group and 21(42%) patients in the control group (*p*<0.05). This study however failed to mention the type of surgery or implant used. But keeping in mind the simplicity, cost effectiveness and technical feasibility of DHS in our set up we presumed that all patients would had been operated with DHS.

In our study we documented hematoma formation, DVT, PE, stroke and mortality in 1(1%) patient each in TXA group (*p*>0.05). Although variable frequency of post-operative complications of TXA have been mentioned in the literature but no increased risk of thromboembolic complications has been reported in meta-analysis. Chen et al.^4^ reported DVT in 10(11.8%) patients, PE in 2 (2.4 %,) stroke in 2(2.4%) and mortality in 5(5.9%) in TXA group but the difference was not statistically significant (*p*>0.05) when compared with placebo of his trial. Zufferey et al^18^ reported adverse vascular events in 16% of his patients in TXA group at 6^th^ weeks postoperatively (*p*>0.05). AlSumadi et al.^19^ reported DVT in 4(1.3%) patients, PE in 2(0.65%) and mortality in 33(10.7%) patients in TXA group (*p*>0.05). Contrary to the above studies Baruah et al.^16^ and Geddes et al.^17^ were of the opinion that TXA is a very safe drug as they had not reported any thromboembolic complications in their TXA intervention group.

### Strength and Limitations of the study

The strength of our trial included an ample sample size, relatively younger age of our patients and matched groups. The limitations of our study were isolated intertrochanteric fracture with DHS surgery only, single dose intravenous preoperative TXA, lack of estimation of peri operative blood loss and a shorter follow up period. We recommend further studies to address all such limitations so that efficacy and safety of TXA is further verified.

## CONCLUSION

Preoperative IV administration of Tranexamic acid (TXA) significantly reduces the frequency of peri operative allogenic blood transfusion in patients undergoing Dynamic Hip Screw (DHS) for intertrochanteric fractures.

### Authors Contribution:

**FAS:** Conceived, designed, data collection and did statistical analysis & editing of manuscript.

**N:** Did data collection and manuscript writing.

**MAA:** Did review and final approval of manuscript and responsible for the accuracy of the work.

**MJI:** Did data collection, Analysis.
